# NLRC5 Deficiency Delays Bone Healing by Inhibiting Osteogenic Differentiation of Bone Marrow-Derived Stem Cells and Altering the Immune Microenvironment

**DOI:** 10.3390/ijms27146489

**Published:** 2026-07-21

**Authors:** Peiying Lyu, Jianru Liu, Yuanbo Wang, Wenyi Liu, Jinsheng Zhong, Xiangying Ouyang

**Affiliations:** Department of Periodontology, Peking University School and Hospital of Stomatology, National Center for Stomatology, National Clinical Research Center for Oral Diseases, National Engineering Research Center of Oral Biomaterials and Digital Medical Devices, Beijing 100081, China

**Keywords:** NLRC5, BMSCs, bone healing, osteogenic differentiation, immune microenvironment

## Abstract

Modulating the immune microenvironment has become an emerging strategy for promoting functional bone regeneration, identifying key therapeutic targets remains challenging. Our previous work showed that Nucleotide-binding oligomerization domain-like receptor family caspase recruitment domain containing protein 5 (NLRC5) is involved in bone destruction associated with periodontitis, but its potential and mechanism in regulating bone tissue repair and regeneration have not been fully elucidated. A monocortical bone defect model was established in the mouse femur to assess the impact of NLRC5 on in situ bone healing and regeneration. Mouse bone marrow-derived mesenchymal stem cells (BMSCs) were isolated to evaluate the effects of NLRC5 on osteogenic differentiation, proliferation, and migration. RNA sequencing was used to explore the direct regulatory mechanism of NLRC5 on osteogenic differentiation of mouse BMSCs. Mass cytometry was employed to examine the effect of NLRC5 on the bone marrow immune microenvironment, followed by in vitro validation experiments. Loss of NLRC5 impaired the healing and regeneration of femoral bone defects in mice, and led to a high inflammatory state in the early stage of healing. The absence of NLRC5 inhibited the osteogenic differentiation ability of BMSCs, and could be restored by NLRC5 overexpression, which was achieved through activation of the phosphatidylinositol 3-kinase/protein kinase B(PI3K/AKT) signaling pathway. Mass cytometry data revealed that NLRC5 may serve as an important factor in maintaining the differentiation and maturation of regulatory T cells (Tregs). By modulating the levels of inflammatory cytokines, NLRC5 further influences the osteogenic differentiation of BMSCs. NLRC5 serves as a key regulator and promising candidate for bone repair. NLRC5 contributes to bone regeneration through a dual mechanism: it promotes BMSCs osteogenic differentiation, at least in part via the PI3K/AKT/β-catenin signaling pathway, and indirectly modulates the local immune microenvironment to facilitate bone repair.

## 1. Introduction

Bone repair is a complex and highly coordinated bioregulatory process involving multiple cell types and associated signaling pathways [1,2]. Stem cells, especially osteogenic stem cells or mesenchymal stem cells, play an important role in bone regeneration given their ability to self-renew and differentiate into osteoblasts [3,4,5]. In addition to the stem cells themselves, immune cell components, such as hematopoietic stem cells, innate immune cells (e.g., macrophages), and adaptive immune cells (e.g., T cells and B cells), are critical for maintaining bone and bone marrow homeostasis [6]. Immunomodulatory factors not only play key roles in bone destruction but also influence bone formation and repair. Nevertheless, the immune modulation of bone formation and repair has not been fully elucidated, and the mechanisms regulating osteogenic cells in the immune microenvironment need more study.

Pattern recognition receptors (PRRs), as the general hub of innate immunity, are involved in a variety of inflammatory diseases [7,8]. Besides participating in immune diseases, PRRs are also associated with bone metabolism [9,10,11]. Nucleotide-binding oligomerization domain-like receptor family caspase recruitment domain containing protein 5 (NLRC5), a PRR, was predominantly expressed in immune cells and was a crucial molecule in regulating inflammatory responses and tumor immunity [12,13]. Interestingly, our group established a periodontitis model in NLRC5-knockout (KO) mice and found that the destruction of alveolar bone was more severe than that in wild-type (WT) mice, more osteoclasts and inflammatory cells in the NLRC5-KO group than in the WT group, suggesting a role for this receptor in inflammatory bone resorption [14]. Furthermore, NLRC5 was reported to regulate T lymphocytes [15,16]. T lymphocytes are an essential component of the body’s immune response and are important for bone immunity [17]. CD4^+^ T cells are key regulators maintaining the osteoclast–osteoblast balance [18]. and CD8^+^ T cells exert an inhibitory effect on osteoclasts [19]. Based on the relationship between NLRC5 and T lymphocytes, we hypothesized that NLRC5 plays a role in immune regulation during bone repair and regeneration. However, there were currently no studies on this matter, and further research was urgently needed.

The phosphatidylinositol 3-kinase/protein kinase B (PI3K/AKT) signaling pathway is mediated by enzyme-linked receptors [20]. This pathway not only participates in the signal transduction of growth factors, cytokines, and extracellular matrix but also regulates multiple cellular functions, such as proliferation, differentiation, and apoptosis [21,22]. Therefore, this pathway is one of the most important in the regulation of stem cell proliferation. NLRC5 has been reported to regulate the proliferation and migration of various cancer cells by regulating the PI3K/AKT signaling pathway [23,24]. However, whether NLRC5 regulates the proliferation and osteogenic differentiation of mesenchymal stromal cells (MSCs) through the PI3K/AKT signaling pathway remains unclear. Therefore, in this study, we aimed to explore the role of NLRC5 in osteogenic formation based on the femoral defect model in NLRC5-KO mice and clarify the mechanisms underlying the effect of NLRC5 on the osteogenic differentiation of stem cells.

## 2. Result

### 2.1. NLRC5 Deficiency Impaired Bone Healing Through Intramembranous Ossification in Femoral Defects of Mice

**Figure 1:**
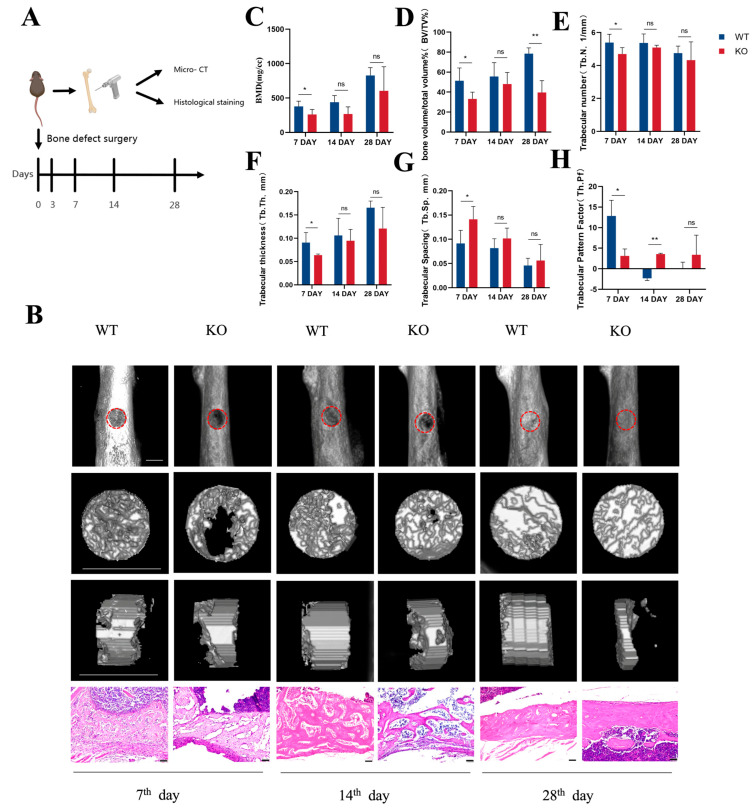
NLRC5 deficiency impairs intramembranous ossification. (**A**) Surgical procedure to create the bone cortical defect and the sampling times. (**B**) Representative 3D images of the injured femur on days 7, 14, and 28 after surgery. The red dotted circle represented the location of the bone defect. (**C**) 3D structural parameters of bone mean density (BMD), (**D**) bone volume/tissue volume (BV/TV), (**E**) trabecular number (Tb.N), (**F**) trabecular thickness (Tb.Th), (**G**) trabecular spacing, and (**H**) trabecular pattern factor (ns: *p* > 0.05; * *p* < 0.05, ** *p* < 0.01). White scale bar: 1 mm, black scale bar: 50 μm. WT, wild type; KO, knockout.

A simplified model of femoral monocortical defects in NLRC5-KO mice was used to investigate the impact of NLRC5 on bone repair. Bone healing was assessed through radiological and histological examinations (Figure 1A). Notably, delayed bone healing was observed in NLRC5-KO mice as depicted in micro-CT images (Figure 1B). Parameters including bone mineral density, bone volume/total volume, trabecular number, and trabecular thickness exhibited significant reductions in the NLRC5-KO group at 7 days postoperatively (Figure 1C–F). Subsequently, at 14 and 28 days, these parameters in the NLRC5-KO group remained lower than those in the WT group, although the disparities were not significant. The trabecular pattern factor, representing the ratio of lamellar to rod-shaped structures, was notably higher in NLRC5-KO mice at 14 and 28 days (Figure 1H), indicating a prevalence of immature bone in the NLRC5-KO group.

On postoperative day 7, WT mice exhibited a significant amount of newly formed woven bone trabeculae at the defect site. The trabeculae were densely packed with osteoblasts, and the surface of the bone defect was covered by multiple layers of spindle-shaped fibroblast-like cells. In NLRC5-KO mice, newly formed trabeculae were also present but showed larger gaps between them, with fewer surrounding cellular components. The defect surface was similarly covered by spindle-shaped fibroblast-like cells. By day 14, the bone trabeculae in WT mice had thickened and increased in number, gradually filling the defect area. Cuboidal osteoblasts were prominently observed within the trabecular gaps. In contrast, NLRC5-KO mice displayed thinner and poorly connected trabeculae with persistent gaps. The gaps contained fewer cells, with an increased presence of lymphocytes. By day 28, bone defects in both groups had largely healed, but mature lamellar bone structures had yet to form.

### 2.2. The Absence of NLRC5 Leads to a Highly Inflammatory Local Microenvironment During the Early Stage of Bone Healing

**Figure 2:**
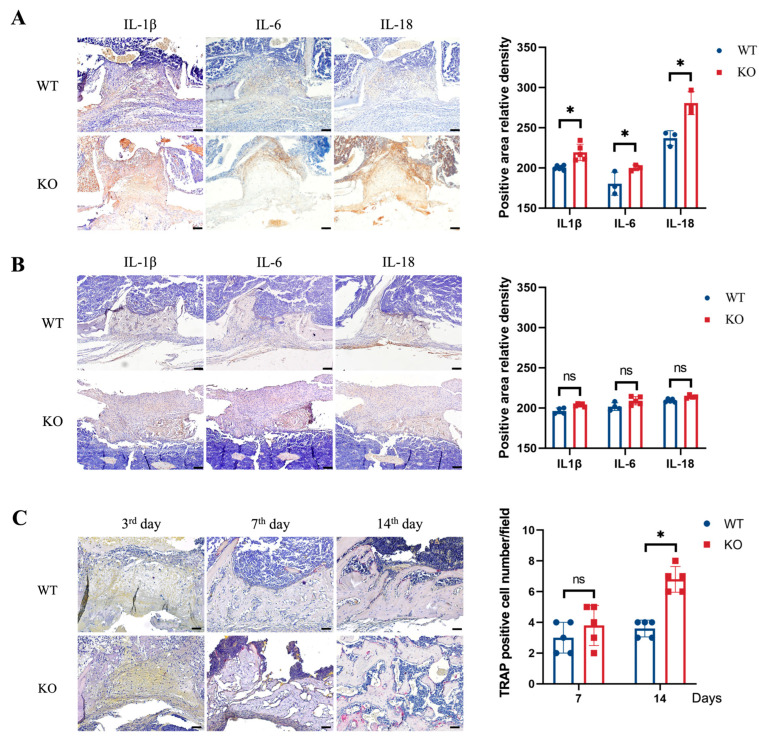
Expression and distribution of IL-1β, IL-6 and IL-18 in the femoral bone defect of mice. (**A**) Images and statistical analysis of IL-1β, IL-6 and IL-18 staining in the femur of WT and NLRC5-KO mice 3 days after bone defect surgery; Scale bar: 100 μm. (**B**) Images and statistical analysis of IL-1β, IL-6 and IL-18 staining in the femur of WT and NLRC5-KO mice 7 days after bone defect surgery; Scale bar: 100 μm. (**C**) Images and statistical analysis of TRAP positive cells; Scale bar: 50 μm. ns: *p* > 0.05; *: *p* < 0.05.

Inflammatory factors IL-1β, IL-6, and IL-18 in the bone defect area of WT and NLRC5-KO mice were assessed via IHC staining on postoperative day 3. As shown in Figure 2A, both groups exhibited significant inflammatory cell infiltration on the medullary side of the granulation tissue. In WT mice, the expression of IL-1β, IL-6, and IL-18 was relatively low and evenly distributed within the granulation tissue. In contrast, NLRC5-KO mice showed markedly higher expression of IL-1β and IL-18, with strong signals observed not only on the medullary side but also throughout the granulation tissue. Quantitative analysis revealed significantly higher inflammatory factor levels in the NLRC5-KO group compared to the WT group (*p* < 0.05).

By postoperative day 7 (Figure 2B), the expression levels of IL-1β, IL-6, and IL-18 had significantly decreased in NLRC5-KO mice. Quantitative analysis showed no significant difference in the levels of inflammatory factors between the two groups (*p* > 0.05), reflecting progress in tissue repair.

As shown in Figure 2C, no osteoclasts were observed at the bone defect sites in either WT or NLRC5 KO mice on postoperative day 3. By day 7, osteoclasts began appearing around the old bone, but their numbers at the defect sites showed no significant differences between the two groups (*p* > 0.05). By day 14, abundant osteoclasts were observed in the gaps between newly formed trabeculae in the NLRC5-KO group, whereas in the WT group, osteoclasts were fewer and limited to areas near trabeculae close to the marrow cavity. Statistical analysis revealed a significant increase in osteoclasts in the NLRC5-KO group compared to the WT group (*p* < 0.05).

The above results indicated that the absence of NLRC5 leads to an abnormal high inflammatory state in the early stage of bone repair. Aberrant immune microenvironment may also contribute to excessive osteoclast activation, which in turn delays bone defect healing. The reasons for this abnormal inflammatory microenvironment still need to be investigated. NLRC5 is an important MHC I class gene product, it plays a significant role in regulating the differentiation and function of immune cells. Therefore, we hypothesized that NLRC5 regulated bone regeneration by influencing the local immune microenvironment in the bone marrow.

### 2.3. NLRC5 Deficiency Compromised the Osteogenic Differentiation of Murine Bone Marrow-Derived Mesenchymal Stem Cells

**Figure 3:**
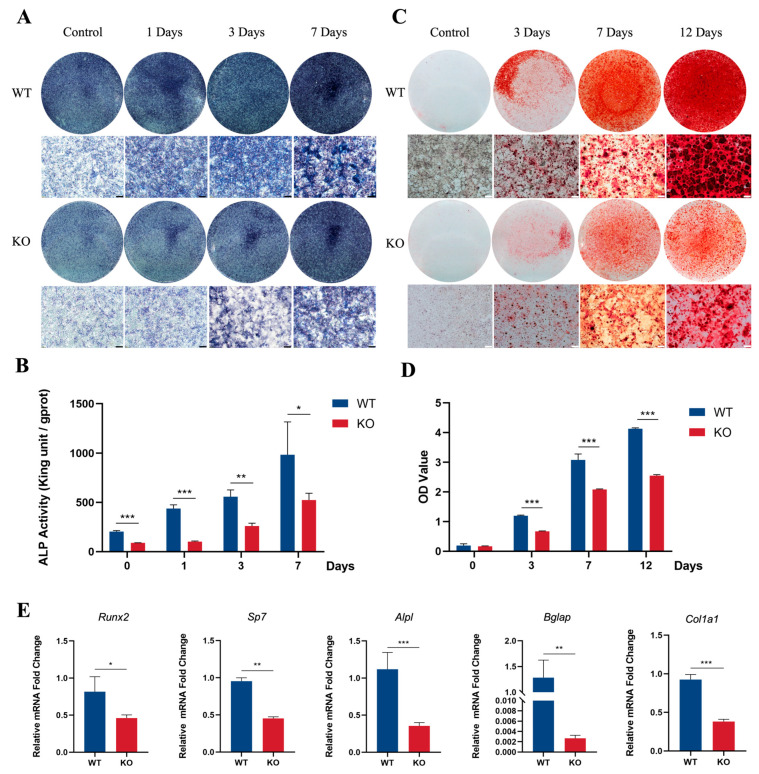
NLRC5 deficiency compromises the osteogenic differentiation ability of BMSCs. (**A**) ALP staining after osteogenic induction, scale bar = 200 μm. (**B**) Quantitative evaluation of ALP activity. (**C**) ARS staining of mineral nodes, scale bar = 200 μm. (**D**) Quantitative evaluation of ARS staining results (* *p* < 0.05, ** *p* < 0.01, *** *p* < 0.001). (**E**) mRNA expression of *Runx2*, *Sp7*, *Alpl*, *Bglap*, and *Col1a1* in the osteogenic differentiation of BMSCs on day 3. ALP, alkaline phosphatase; ARS, alizarin red S; WT, wild type; KO, knockout; BMSCs, murine bone marrow-derived mesenchymal stem cells.

To investigate the mechanism underlying the action of NLRC5 in bone regeneration, the effect of NLRC5 on the proliferation, migration, and osteogenic differentiation of mouse bone marrow-derived mesenchymal stem cells (BMSCs) was examined in vitro. BMSCs were isolated from the lower limb bones of 2-week-old mice. No significant differences in the proliferation rates and migration of BMSCs were observed between the NLRC5-KO and WT groups (Supplementary Materials S1). However, it was noted that the NLRC5-KO group exhibited significantly lower ALP activity and less mineralized nodules (Figure 3A–D). Furthermore, upon NLRC5-KO, there was a marked decrease in the mRNA expression of key osteogenic genes, such as *Runx2*, *Sp7*, *Alpl*, *Bglap*, and *Col1a1* (Figure 3E).

Subsequently, NLRC5 expression was rescued in BMSCs via viral vector, and higher ALP activity and increased mineralized nodules were observed (Figure 5C). We also recovered the mRNA expression of osteogenic genes (Figure 5D). These results reinforce the notion that NLRC5-KO significantly compromises the osteogenic differentiation potential of BMSCs, with the subsequent reinstatement of osteogenic capacity upon the rescue of NLRC5 expression.

### 2.4. NLRC5 Regulates mBMSC Osteogenic Differentiation via the PI3K/AKT/GSK3β/β-Catenin Signaling Pathway

**Figure 4:**
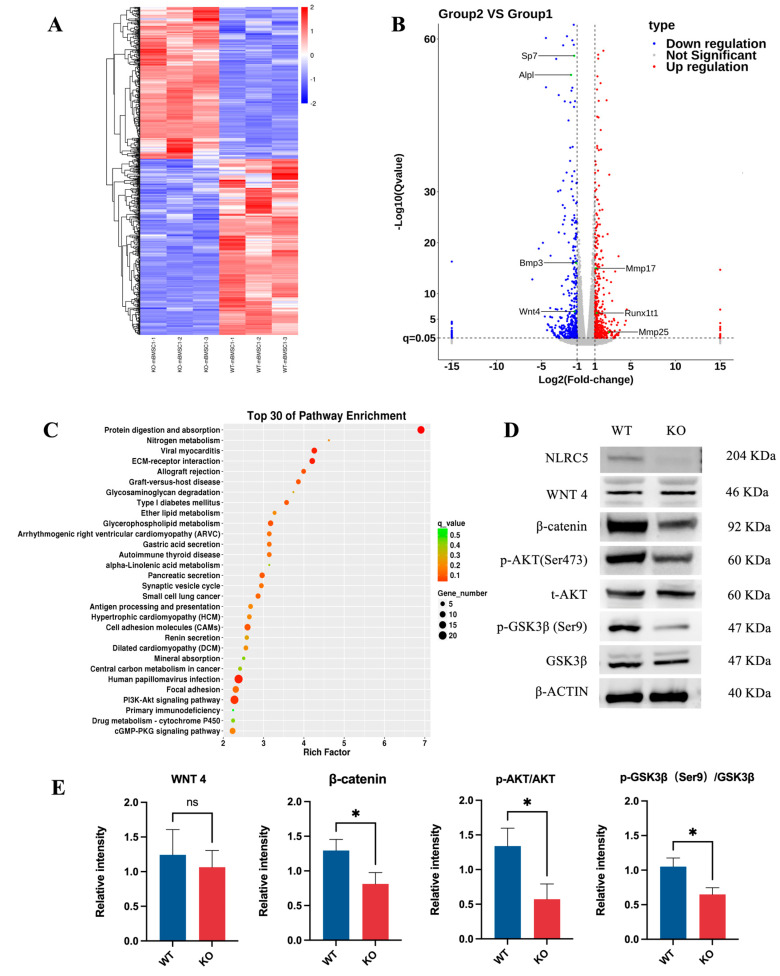
Comparison of BMSCs from NLRC5-KO and WT groups using transcriptomic analysis. (**A**) Heatmap showing differentially expressed genes. Blue and red represent low and high expression values, respectively. (**B**) Upregulated and downregulated bone metabolism-related genes. (**C**) KEGG functional enrichment analysis. (**D**) Immunoblot images and (**E**) statistical analysis showing the effect of NLRC5-KO on the expression of the Wnt4/β-catenin, p-AKT/AKT, and p-GSK3β/GSK3β, ns: *p* > 0.05; *: *p* < 0.05. KEGG, Kyoto Encyclopedia of Genes and Genomes; WT, wild type; KO, knockout; BMSCs, murine bone marrow-derived mesenchymal stem cells.

**Figure 5:**
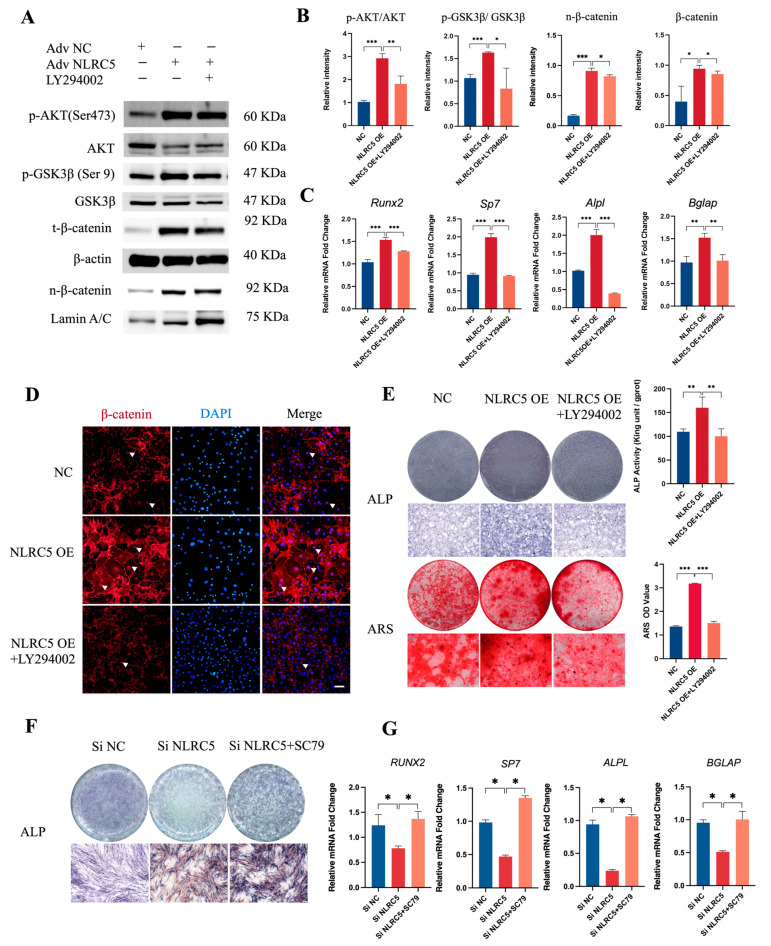
NLRC5 regulates mBMSC osteogenic differentiation through the PI3K/AKT/GSK3β/β-catenin signaling pathway. (**A**) Protein expression levels and statistical analysis (**B**) of p-AKT/AKT, p-GSK3β/GSK3β, β-catenin (nuclear), and β-catenin (total) in NLRC5-KO BMSC NC, NLRC5 OE, or NLRC5 OE treated with LY294002 (an inhibitor of PI3K). (**C**) mRNA expression levels of *Runx2*, *Sp7*, *Alpl* and *Bglap* during the osteogenic differentiation of BMSCs on day 3 in the different groups. (**D**) Immunofluorescence staining results of catenin. The red fluorescence represents β-catenin, the blue fluorescence indicates the nucleus, and the arrow shows β-catenin inside the nucleus. (**E**) Alkaline phosphatase activity (ALP) and alizarin red S (ARS) staining of NLRC5-KO BMSCs from different groups. (**F**) ALP staining of human BMSCs from different groups. (**G**) mRNA expression levels of *Runx2*, *Sp7*, *Alpl*, and *Bglap* during the osteogenic differentiation of human BMSCs on day 3 in the different groups. scale bar = 200 μm. (* *p* < 0.05, ** *p* < 0.01, *** *p* < 0.001). BMSCs, murine bone marrow-derived mesenchymal stem cells; NC, vector negative control group; OE, overexpression group; Si, small interfering RNA.

To elucidate the molecular mechanism underlying NLRC5’s promotion of osteogenic differentiation, an RNA-seq analysis was conducted using three biological replicates of WT and NLRC5-KO BMSCs. The results, as illustrated in Figure 4A, unveiled 303 upregulated and 328 downregulated differentially expressed genes between NLRC5-deficient and WT BMSCs. Particularly noteworthy is the downregulation of key osteogenesis-related genes *Bmp3*, *Alpl*, and *Sp7* (Osterix), alongside the upregulation of osteoclast-related genes such as *Mmp7*, *Mmp25*, and *Runx1t1* (Figure 4B). KEGG enrichment analysis (Figure 4C) highlighted a significant decline in the PI3K/AKT signaling pathway within the NLRC5-KO group. Then, Western blot results identified a decrease in the protein levels of phosphorylated AKT (Ser473), GSK3β (Ser9), and β-catenin in BMSCs from the NLRC5-KO group compared to those from the WT group (Figure 4D). Restoring the expression of NLRC5 in NLRC5-KO BMSCs reversed the levels of AKT (Ser473), GSK3β (Ser9), total β-catenin, and total nuclear β-catenin (Figure 5A,B). These findings suggested that NLRC5 might operate through the PI3K/AKT/β-catenin signaling pathway in BMSCs.

Subsequently, the PI3K/AKT signaling pathway was inhibited by the PI3K inhibitor LY294002 in NLRC5-rescued BMSCs, leading to the inhibition of AKT and GSK3β signal transduction, as well as a decline in total and nuclear β-catenin levels (Figure 5A). The immunofluorescence staining results shown in Figure 5B indicated that following NLRC5 overexpression, the red fluorescence intensity in the nucleus significantly increased compared with the NC group, suggesting enhanced nuclear translocation of β-catenin. Upon treatment with LY294002 to inhibit AKT pathway activity, the red fluorescence intensity in the nucleus decreased, indicating reduced nuclear translocation of β-catenin. These findings further demonstrate that NLRC5 can influence β-catenin expression and nuclear translocation by regulating AKT pathway activity, thereby modulating its downstream effector molecules. This PI3K/AKT inhibitor also resulted in reduced ALP activity and mineralization ability of the BMSCs (Figure 5D), accompanied by decreased expression levels of osteogenic genes *Runx2*, *Sp7*, *Alpl*, and *Bglap* (Figure 5C).

Human BMSCs with NLRC5 knocked down using siRNA were used to mimic NLRC5-KO mouse BMSCs [25]. After knockdown, SC79 was used to activate the AKT pathway, and ALP activity and osteogenic gene expression were assessed following osteogenic induction. The results showed that NLRC5 knockdown led to reduced ALP activity and decreased mRNA expression of osteogenic genes. Upon treatment with the AKT pathway agonist SC79, ALP activity increased, and the expression levels of osteogenic genes were restored. These findings indicate that the AKT pathway activator can rescue the impaired osteogenic differentiation caused by NLRC5 knockdown, demonstrating that NLRC5 functions upstream of the PI3K/AKT pathway.

In conclusion, NLRC5 deficiency impedes the osteogenic differentiation of BMSCs by the PI3K/AKT/GSK3 β/β-catenin signal pathway.

### 2.5. NLRC5 Deficiency Impeded CD4^+^ T Cells Differentiating into Treg Cells and Subsequently Affecting the Osteogenic Differentiation of BMSCs

**Figure 6:**
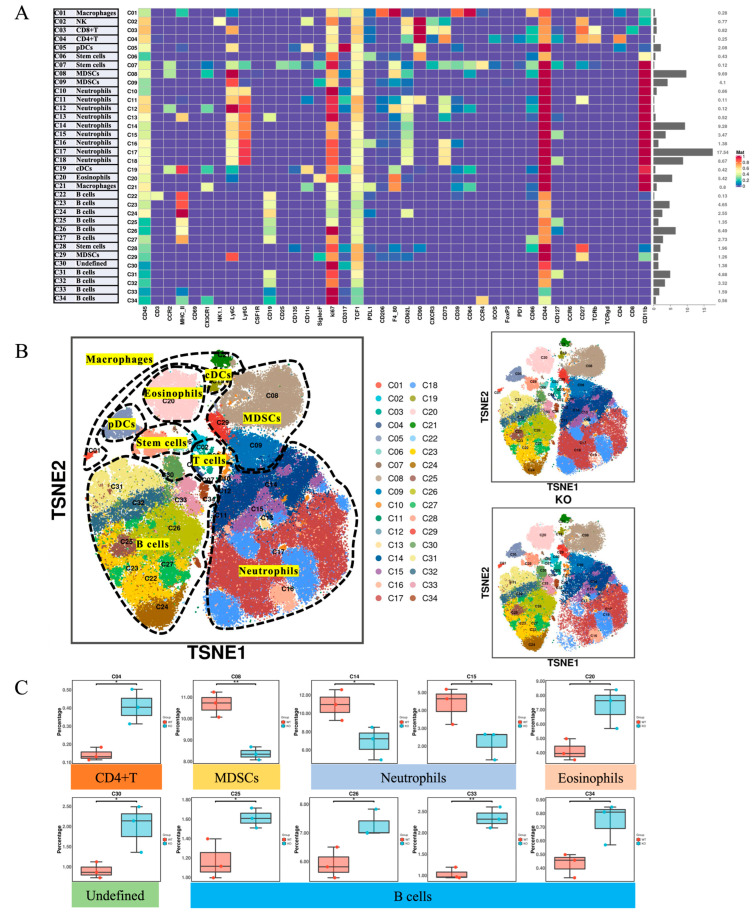
The effect of NLRC5 on the distribution of immune cell subpopulations in mouse bone marrow. (**A**) Unsupervised clustering algorithm analyzes the heatmap. (**B**) T-SNE cell dimensionality reduction clustering shows the differential cell subpopulations. (**C**) Comparison of differential immune cell subpopulations in bone marrow of WT and NLRC5-KO mice. *: *p* < 0.05, **: *p* < 0.01.

As shown in Figure 6A, unsupervised clustering of CD45-positive bone marrow cells identified 34 distinct subpopulations, representing major immune lineages such as T lymphocytes, B lymphocytes, natural killer (NK) cells, neutrophils, monocytes/macrophages, dendritic cells, and hematopoietic stem cells. Figure 6B uses t-SNE-based dimensionality reduction with heatmap visualization, highlighting clear inter-cluster separation with color-coded cluster identities for improved interpretation.

Statistical analysis of cell counts differences among subpopulations revealed ten significantly altered subgroups across six major cell types (adjusted *p* < 0.05). Figure 6C highlights the following significant changes in NLRC5-KO mice compared to WT controls: (i) an increased proportion of CD4^+^ T cells (TCRβ^+^CD4^+^CD27^+^CD73^+^CD90^+^) in bone marrow (*p* < 0.05); (ii) a marked decrease in myeloid-derived suppressor cells (MDSCs; CD11b^+^Ly-6C^+^Ly-6G^−^F4/80^+^) (*p* < 0.01); (iii) reduced frequencies of neutrophils defined by two phenotypes—CD11b^+^Ly-6C^+^Ly-6G^+^ and CD11b^+^Ly-6C^+^Ly-6G^+^CD127^+^ (both *p* < 0.05); (iv) elevated eosinophil (CD11b^+^SiglecF^+^F4/80^+^) proportions (*p* < 0.05); and (v) a significant expansion of B cell subpopulations (*p* < 0.05) [26], comprising four discrete subsets: two immature B cell clusters (CD19^+^CD44^+^CD127^+^MHC II^+^), one activated B cell cluster (CD19^+^CD44^+^MHC II^+^), and one CX3CR1+ B cell subset associated with lymphoma progression and metastasis (CD19^+^CX3CR1^+^CCR4^+^MHC II^+^) [27,28]. No significant differences were observed in the frequencies of monocytes/macrophages, dendritic cells, or CD8^+^ T cell subpopulations.

These findings demonstrate that NLRC5, acting as a transcriptional coactivator for MHC class I genes, plays a key role in modulating innate immune cell composition (e.g., neutrophils, eosinophils, MDSCs) and indirectly influences adaptive immunity. Disruption of innate immune cell homeostasis can impair antigen presentation, affecting the development, distribution, and functional capacity of adaptive immune cells. NLRC5 deficiency profoundly alters the immune landscape in the bone marrow, potentially impacting immune–osteolineage communication essential for bone homeostasis, formation, and repair.

Transcriptomic analysis of BMSCs revealed enrichment for processes related to T cell receptor signaling and ligand recognition. CyTOF immunophenotyping showed a majority of B cells that were phenotypically immature precursors with limited effector functions. Subsequently, further analysis focused on CD4^+^ T lymphocyte heterogeneity. These cells differentiate into subsets such as Th1, Th2, Th9, Th17, Th22, follicular helper T (Tfh), and regulatory T (Treg) cells upon activation in cytokine-driven environments [29].

**Figure 7:**
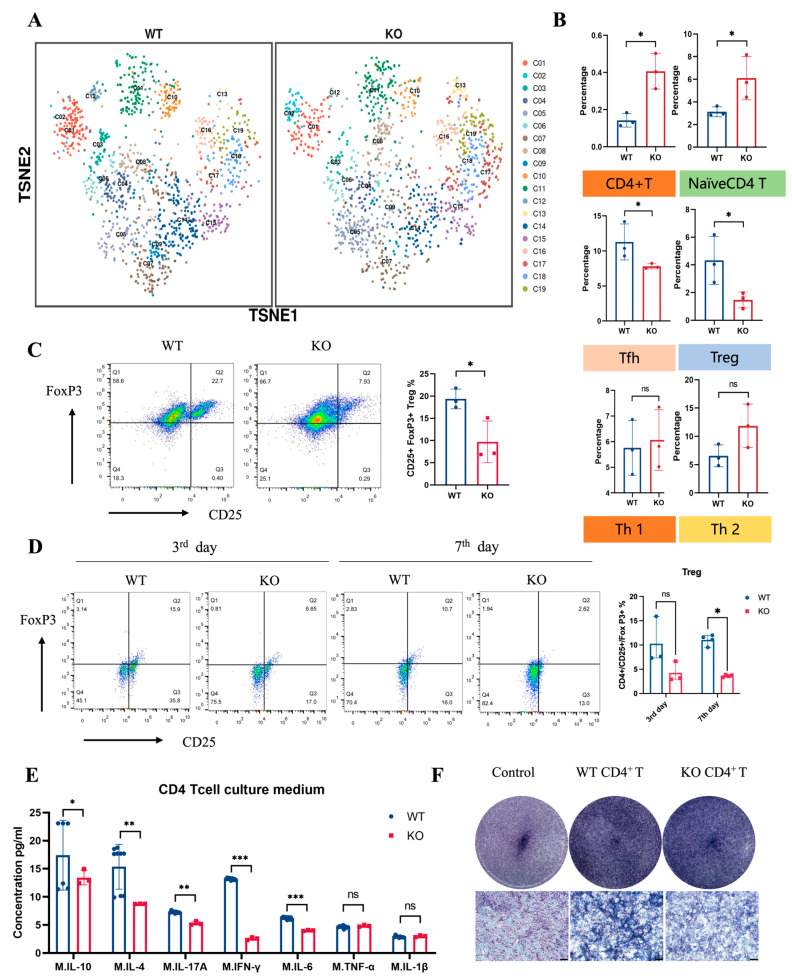
Effect of NLRC5 on the proportion of immune cell subsets in bone marrow. (**A**) T-SNE is used to perform cell dimensionality reduction and clustering to display the differences in CD4 T cell subpopulations. (**B**) The proportion of total CD4^+^T cells (TCRβ ^+^CD4^+^CD27^+^ CD73 ^+^CD90^+^) in the bone marrow of NLRC5-KO mice; the proportion of Naïve CD4 T cells (CD4^+^ CD44^low^ CD62L^hi^), Treg cells (CD4^+^ CD25^+^FoxP3^+^ CD44^+^ CD62L^−^), Tfh cells (CD4^+^ ICOS^+^ PD1^+^), Th1 (CD4^+^CXCR3^+^) and Th2 (CD4^+^CCR4^+^CXCR3^−^) in the NLRC5-KO group and WT group. (**C**) The proportion and statistics of Treg cells (CD4^+^ CD25^+^FoxP3^+^) induced in vitro. (**D**) Flow cytometric analysis of the femoral defect tissues at postoperative days 3 and 7. (**E**) Effect of NLRC5 on the secretion of inflammatory cytokines by CD4^+^T cells in mice. (F) Effect of WT and NLRC5-KO CD4^+^T cells on ALP activity of BMSCs, scale bar = 200 μm. WT, wild type; KO, knockout; BMSCs, murine bone marrow-derived mesenchymal stem cells; * *p* < 0.05, ** *p* < 0.01, *** *p* < 0.001.

Re-clustering of CD4+ cells yielded 19 transcriptionally and phenotypically resolved subpopulations (Figure 7A). Compared to WT mice (Figure 7B), NLRC5-KO mice exhibited an increased proportion of overall CD4+ T cells in bone marrow (TCR β^+^CD4^+^CD27 ^+^ CD73^+^CD90^+^) (*p* < 0.05); however, the proportions of regulatory T cells (CD4^+^ CD25^+^ FoxP3^+)^ and Tfh cells (CD4^+^ ICOS^+^ PD1^+^) decreased in the NLRC5-KO group compared to those in the WT group (*p* < 0.05); the proportion of Naïve CD4^+^ T cells (CD4^+^ CD44^low^CD62L^hi^) increased (*p* < 0.05). The proportion of Th1 cells (CD4^+^CXCR3^+^) and Th2 cells (CD4^+^ CCR4^+^ CXCR3^−^) in the bone marrow of NLRC5-KO mice slightly increased, but the difference was not significant (*p* > 0.05).

Flow cytometric analysis of femoral defect tissues on postoperative days 3 and 7 showed a significant reduction in Treg cell proportions in NLRC5-KO mice compared to WT controls (Figure 7D). These results align with CyTOF findings from bone marrow, indicating decreased local Treg accumulation in NLRC5-deficient mice during the early stages of bone healing. These findings provided direct evidence that NLRC5 deficiency impairs Treg cell recruitment at the site of injury.

To further validate the role of NLRC5 in Treg cell differentiation, CD4^+^T cells were extracted from mouse spleen cells and induced into Treg cells. The results showed that, compared to that in the WT mice, the proportion of induced Treg cells (CD4^+^ CD25^+^FoxP3^+^) in the NLRC5-KO group decreased (*p* < 0.05) (Figure 7C), with lower expression levels of IL-10, IL-4, IL-17A, IFN-γ, and IL-6 in the supernatant (Figure 7E).

NLRC5-KO BMSCs were incubated with the supernatant of the WT or NLRC5-KO Treg cells and then allowed to undergo osteogenic differentiation for 7 days. The results showed that ALP activity in the NLRC5-KO group was lower than that in the WT group (Figure 7F), suggesting that NLRC5 deficiency inhibited the osteogenic differentiation of BMSCs indirectly by altering the local immune microenvironment.

## 3. Discussion

The study revealed that the innate immune molecule NLRC5 serves as an indispensable regulator in bone repair and regeneration. This receptor not only directly regulated osteogenic differentiation of BMSCs and the expression of osteogenic-related factors at least in part through the PI3K/AKT/GSK3β/β-catenin signaling pathway, but also affected the differentiation of CD4^+^T cells and the expression of various inflammatory cytokines, which indirectly influenced the osteogenic differentiation of BMSCs. Our previous study showed that NLRC5 plays a role in bone destruction induced by inflammation [14]. These findings together indicate that NLRC5 might be a crucial immune switch for bone metabolism. This study represents a breakthrough in our understanding of the relationship between PRRs and bone healing and regeneration. Our findings also offer insights into potential therapeutic targets for the development of clinical treatments.

In this study, a single cortical bone defect model was established in NLRC5-KO mice to investigate the role of NLRC5 in intramembranous bone regeneration. Mesenchymal stem cells from periosteum and endosteum underwent intramembranous ossification for subsequent bone repair [30,31]. During the synthetic metabolic modeling phase, the lack of cuboidal osteoblasts around the new bone trabeculae in NLRC5-KO mice was observed. In the degradative metabolic remodeling phase, more osteoclasts were found around bone trabeculae in NLRC5-KO mice, indicating an increase in osteoclast numbers and bone resorption at this stage. By combining these two processes, we could explain why the neoformed bone trabeculae in the bone defects of NLRC5-KO mice appeared thin with enlarged intertrabecular spaces and delayed bone healing. This phenomenon was similar to the bone healing process observed in RAG1-KO mice, characterized by a lack of mature T and B lymphocytes [32]. Decreased bone formation, increased osteoclast numbers, and reduced bone strength of newly formed bone were shown post-fracture in RAG1-KO mice. Therefore, the effect of NLRC5 on lymphocytes was explored in the present study.

A close relationship between NLRC5 and CD8^+^ T cells has been reported [15]. The present study revealed for the first time, to our knowledge, that NLRC5 also regulates CD4^+^ T cells. CD4^+^ T cells, known as helper T cells, can differentiate into various subtypes such as Th1, Th2, Th17, Treg, Tfh, Th9, and Th22 [32]. Compared to WT mice, NLRC5-KO mice showed an increased total number of CD4^+^ T cells in the bone marrow, with a decrease in the proportion of Treg and Tfh cells and an increase in the proportion of naïve CD4^+^T cells. These results indicated that NLRC5 deficiency hindered the activation of CD4^+^ T cells and led to abnormal CD4^+^ T cell function. Additionally, this study showed that the absence of NLRC5 inhibited the differentiation of CD4^+^ T cells into Treg cells. Treg cells are a subset of T cells with immunosuppressive functions capable of secreting cytokines IL-10 and TGF-β, maintaining immune tolerance and homeostasis [33]. IL-10 inhibits the expression of IL-6, TNF-α, RANKL, and RANK, thereby inhibiting osteoclast maturation and differentiation [34]. Additionally, IL-10 could hinder osteoblast apoptosis and upregulate the expression of osteocalcin, osteopontin, and ALP in osteoblasts [35]. Therefore, Treg cells play a vital positive role in bone healing and regeneration. Zaiss et al. found that transgenic mice with elevated levels of Treg cells exhibited higher bone density and lower long bone resorption compared to WT mice [36]. In the present study, the lack of NLRC5 inhibits the differentiation of CD4^+^ T cells into Treg cells, resulting in decreased expression of anti-inflammatory factors such as IL-10 and IL-4, thereby indirectly suppressing the osteogenic differentiation of BMSCs. These findings demonstrated the critical role of NLRC5 in maintaining the immune microenvironment of the bone marrow, exerting a significant effect on bone healing and regeneration by regulating the functional machinery of immune cell subsets.

CyTOF analysis revealed changes in other immune cell populations in the bone marrow of NLRC5-KO mice. Recent evidence highlighted the critical role of eosinophils in bone metabolism, primarily by inhibiting osteoclast differentiation to prevent excessive bone resorption [37]. However, their contribution to acute bone defect healing remains largely unexplored, and the underlying mechanisms are not yet fully understood.

Neutrophils are key responders during the very early stages of fracture healing (24–72 h) [38]. They contribute to host defense by phagocytosing necrotic tissue and pathogens and releasing reactive oxygen species (ROS) and protease [39,40]. However, the excessive release of these substances could impair bone regeneration, and their prolonged presence has been shown to inhibit the mineralization of BMSCs [41]. Neutrophil depletion has been reported to disrupt bone healing and alter the inflammatory response [42], underscoring their indispensable role in the early phases of repair.

B cells play multifaceted roles in bone metabolism. By producing osteoprotegerin, B cells counteract RANKL and promote bone regeneration [43]. However, under pathological conditions such as rheumatoid arthritis, B cells can inhibit the osteogenic differentiation of MSCs via CCL3/TNF and ERK/NF-κB pathways [44]. Additionally, during estrogen deficiency, B cell-derived RANKL enhances osteoclast activity [45].

MDSCs emerge during the transition from the inflammatory to the repair phase of bone healing. They maintain immune homeostasis by suppressing excessive inflammation and can directly contribute to bone formation by expressing Runx2 and osteocalcin [46,47]. MDSCs can also differentiate into mature osteoclasts and indirectly promote bone resorption by suppressing T cell production of cytokines such as IFN-γ, IL-4, and IL-10 [48,49]. Additionally, MDSCs facilitate angiogenesis by producing VEGF-A, bFGF, and MMP-9. Their removal has been shown to impair healing, while adoptive transfer accelerates it [50,51]. How NLRC5 affects the functions of these immune cells to regulate bone defect healing remains to be explored in future work.

BMSCs play a pivotal role in bone repair processes [52]. In NLRC5-KO mice, impaired osteogenic ability of BMSCs and altered secretion of osteogenic-related proteins were observed. During this process, the PI3K/AKT/β-catenin pathway plays a crucial role. The PI3K/AKT signaling pathway is essential in controlling cell survival, growth, and differentiation [21]. Activation of the AKT signal facilitated MSC differentiation into osteoblasts. In addition, activated PI3K/AKT directly promoted FoxOs expression, thereby regulating bone formation [53]. Mice treated with the PI3K inhibitor LY294002 showed impaired bone healing, indicating a positive regulatory role for this signaling pathway in bone repair [54]. The β-catenin signaling pathway was also crucial in skeletal development and repair [55]. Interestingly, this study showed that NLRC5 activated the β-catenin pathway not by Wnt ligands but by regulating the transcription of GSK-3β through the PI3K/AKT signaling pathway. This finding confirmed the crosstalk between the PI3K/AKT and β-catenin signaling pathways, which was in accordance with previous research [54,56]. The relationship between NLRC5 and these two vital osteogenic pathways demonstrated its essential role in BMSCs osteogenic differentiation. Therefore, enhancing the expression of NLRC5 in human BMSCs may present a new direction for promoting bone healing and regenerative therapies.

Several limitations of the present study should be acknowledged. The mouse model used in the experiments was a global NLRC5 knockout, making it impossible to independently evaluate the specific roles of BMSCs, immune cells, or systemic factors in femoral defect repair. Additionally, due to the technical challenges in constructing an adeno-associated virus (AAV) for NLRC5 overexpression, the study lacks in vivo rescue experiments for validation. Further experiments using conditional knockout or bone marrow chimeric mice will be performed to address these limitations and strengthen our mechanistic conclusions.

## 4. Materials and Methods

### 4.1. Generation of NLRC5-KO Mice

The NLRC5 gene knockout mice were purchased from Gene Pharma Technology (Nanjing, Jiangsu, China). The wild-type mice were obtained from Beijing Vital River Laboratory Animal Technology Co., Ltd. (Beijing, China). Mice in both groups were C57BL/6J inbred strains.

### 4.2. Femoral Monocortical Defect Model

Femoral monocortical defect modeling was performed using 8-week-old male mice. Anesthesia and euthanasia were achieved by administering tribromoethanol sodium. Femoral monocortical defects (1.0 mm diameter) were created on the anterior surface of the distal femur using a round bur under saline irrigation. Drilling was terminated upon loss of resistance to avoid contralateral cortical damage. The muscle layer was closed with 5-0 absorbable sutures, and the skin was closed with non-absorbable sutures. All procedures were performed under aseptic conditions in an SPF facility. Both WT and NCLR5-KO mice were included in this study and sacrificed on days 3, 7, 14 or 28 after surgery. Femoral samples were harvested for subsequent histological examination and imaging analysis.

### 4.3. Micro-Computed Tomography Analysis

micro-CT was used to scan the harvested femurs from WT and NLRC5-KO mice (n = 5), the scanning parameters and reconstruction analysis methods were the same as those described in our previous study [25].

### 4.4. Histological Analysis

The mouse femurs were fixed and decalcified for 2 weeks. Prepared 5 µm thick paraffin sections and performed hematoxylin and eosin (HE) staining (Solarbio, Beijing, China, #G1120) and TRAP staining solution (#387A, Sigma, Saint Louis, MO, USA).

### 4.5. Isolation and Culture of BMSCs

Briefly, the femur and tibia of 2-week-old mice were dissected, and bilateral epiphyses were excised. Subsequently, the bone marrow tissue was flushed out using a pre-cooled specialized medium for MSCs (ScienCell, San Diego, CA, USA, #7501). The obtained cell suspension was then cultured in a humidified incubator at 37 °C under 5% CO_2_ for 24 h. Following two consecutive passages, isolated BMSCs were utilized for subsequent experiments.

### 4.6. Cell Osteogenic Induction and Staining

When BMSCs growth reached 70–80% of confluence, the medium was changed to the osteogenic induction medium [25]. After osteogenic induction, the cells were stained by alkaline phosphatase (ALP) staining and alizarin red staining (ARS). The intracellular ALP activity was quantified as before [25].

### 4.7. Real-Time PCR

Total RNA was extracted from the cells using *SteadyPure* Rapid RNA Extraction Kit (Accurate Biotechnology, Changsha, Hunan, China, #AG21023) [57]. cDNA synthesis and real-time PCR were performed using the kits from ABclonal (Wuhan, China, #PK20429 and #RK21203) as before [58]. The primer sequences are shown in Table 1. Normalization of all data was carried out based on β-actin expression. Relative gene expression levels were determined using the 2^−ΔΔCT^ method.

### 4.8. RNA -Seq and Bioinformatics Analysis

High-throughput sequencing performed by Biotechnology Corporation (Shanghai, China). Total RNA was extracted from NLRC5-KO and WT BMSCs (n = 3). Significant differentially expressed genes (DEGs) were identified using edgeR software 3.40.2, with a threshold of |fold change| > 2 and a false discovery rate (FDR) < 0.05. GO enrichment and KEGG analyses were further performed to clarify the biological significance of the differentially expressed genes.

### 4.9. Western Blotting

In accordance with the manufacturer instructions, protein lysis buffer (Solarbio, #R0010) containing a protease inhibitor cocktail (Huaxingbio, Beijing, China, #HX1863) was used to extract total cell protein, and a nuclear protein extraction kit (YEASEN, Shanghai, China, #20126ES50) was used to extract nuclear protein. Protein bands were viewed and quantified using ImageJ software 1.53c (National Institutes of Health, Bethesda, MD, USA). The following primary antibodies were employed, all at a dilution of 1:1000: anti-β-actin (ZSGB-BIO, Beijing, China, #TA-09), anti-NLRC5 (ABclonal, Wuhan, Hubei, China, #A16740), anti-WNT4 (ABclonal, #A7809), anti-AKT and anti-p-AKT (Cell Signaling Technology, Danvers, MA, USA, #4691 and #9271), anti-GSK3β and anti-p-GSK3β (ZENBIO, Durham, NC, USA, #221162 and #R22943), anti-LaminA/C (ZENBIO, #R26947), and anti-β-catenin (Proteintech, Wuhan, China, #51067-2-AP).

### 4.10. Adenovirus Transfection and PI3K/AKT Signaling Pathway Inhibition

The full-length NLRC5 cDNA and negative control vectors were obtained from XIEBHC BIO (Beijing, China). BMSCs were infected with adenovirus at a multiplicity of infection of 100:1 in the presence of polybrene (Gene Pharma) at a concentration of 5 μg/mL. After 24 h, fresh medium was used to replace the culture medium containing the virus. The cells were utilized for subsequent experiments after transfection with the virus for 48 h. To inhibit PI3K/AKT signaling, BMSCs were treated with LY294002 (Selleck, Houston, TX, USA, #S1105), a PI3K inhibitor, at a concentration of 3 μM. To active PI3K/AKT signaling, BMSCs were treated with SC79 (MedChemExpress, South Brunswick Township, NJ, USA, #HY-18749) of 10 μM. Cells pretreated for 24 h were employed for follow-up experiments.

### 4.11. Immunofluorescence Staining

Cells were fixed and permeabilized with 0.1% Triton X-100. Cells were blocked with 5% BSA for 60 min. Subsequently, cells were incubated with anti-β-catenin (Proteintech) overnight at 4 °C. The sections were incubated with the fluorescent secondary antibody (ABclonal, #AS039), then mounted and photographed.

### 4.12. CyTOF Analysis

Bone marrow flushed cells were collected from 4-week-old WT and NLRC5-KO mice. The subsequent CyTOF experiments were commissioned to Zhejiang Puluoting Health Technology Co., Ltd. (Hangzhou, China) and performed according to the procedures described in previous study (Supplementary Material S2) [59,60]. The antibody list can be found in Supplementary Material S3.

### 4.13. Sorting of CD4 Positive T Cells

Mice were anesthetized with excessive pentobarbital sodium, and the spleen was extracted. The splenocyte suspension was obtained by grinding and sieving. Erythrocyte lysate was added, and the sample was lysed on ice for 15 min. The cell precipitation after centrifugation was resuspended in 2% FBS PBS buffer; then, CD4 antibody (Biolegend, San Diego, CA, USA, #100405) was added, and the sample was incubated at 4 °C for 30 min without light. The cells were sorted on a FACS Aria III flow cytometer to collect CD4 positive cells.

### 4.14. Induction of Treg Cells

The sorted CD4 positive cells were centrifuged, and the cell precipitation was resuspended in 1640 medium containing 10% FBS. The cell density was adjusted to 1 × 10^6^ cells/mL, and the sample was placed in a 24-well plate for further culture. To induce Treg cells, CD3 (final concentration 5 μg/mL) and CD28 (final concentration 2 μg/mL) co-stimulatory molecules were added to each well to activate T cells, and then IL-2 (final concentration 20 ng/mL) and TGF-β (final concentration 5 ng/mL) were added for 4 days to induce Treg cell differentiation. The cell supernatant was absorbed for cytokine detection, and the CD4^+^T cells induced were identified through flow cytometry to determine the proportion of Treg cells. CD3 (#100301), CD28 (#102101), and TGF-β (#763104) were obtained from Biolegend, and IL-2 (#212-12) was obtained from PeproTech (Cranbury, NJ, USA).

### 4.15. Flow Cytometry for Identifying Treg Cells

After induction, the cell precipitation was washed and resuspended in PBS buffer. The sample was stained with CD25 antibody (Biolegend, #102011). Then, the cells were fixed and the membrane was broken using 0.1% Triton X-100. Finally, FoxP3 antibody (Biolegend, #126403) was added and incubated. Cells were detected using a Cytoflex flow cytometer (Beckman Coulter, Inc., Brea, CA, USA).

### 4.16. Multi-Factor Detection of Cell Supernatant

This part was entrusted to ABclonal for detection. The expression of interleukin-10 (IL-10), IL-4, IL-17A, interferon-γ (IFN-γ), IL-6, tumor necrosis factor-α (TNF-α), and IL-1β in the supernatant of CD4^+^T cells of WT mice and NLRC5-KO mice was detected.

### 4.17. Indirect Co-Culture of BMSCs and CD4^+^T Cells

NLRC5-KO BMSCs were placed in 24-well plates. After the cells grew to 70–80%, the complete medium was replaced with the conditioned medium for the differentiation of WT and NLRC5-KO Tregs. After 24 h of culture, the medium was replaced with osteogenic induction medium. After 7 days of osteogenic induction, ALP staining was performed, and photographs were taken under the microscope.

### 4.18. Statistical Analysis

Data are presented as mean ± standard deviation. Each dataset was assessed for homogeneity, and the results were analyzed using one-way ANOVA or unpaired Student’s *t*-test. For data that did not follow a normal distribution, the Mann–Whitney U test or Kruskal–Wallis test was used. A *p*-value of <0.05 was considered significant. All statistical analyses were conducted using SPSS software (version 23.0; IBM Corp., Armonk, NY, USA). All experiments were repeated three or more times. NS indicates no significant difference.

## 5. Conclusions

We demonstrated that NLRC5 works as a pivotal immunoregulatory molecule in bone healing and regeneration for the first time, to our knowledge. NLRC5 directly regulated the osteogenic differentiation of BMSCs at least in part through PI3K/AKT/β-catenin signaling pathways. It also modulated the immune microenvironment by regulating the differentiation of CD4+ T cell, thereby indirectly affecting osteogenic differentiation of BMSCs. Given this crucial role of NLRC5 in bone regeneration, it might serve as a target for future therapeutic interventions.

## Figures and Tables

**Table 1:** Sequences of primers used for real-time PCR.

Genes	Primer Sequences (5′ to 3′)
*β-actin*	Forward: GGCTGTATTCCCCTCCATCG Reverse: CCAGTTGGTAACAATGCCATGT
*Runx2*	Forward: GAGGCCGCCGCACGACAACCG Reverse: CTCCGGCCCACAAATCTCAGA
*Sp7*	Forward: ATTCTCCCATTCTCCCTCCCT Reverse: GGAAGGGTGGGTAGTCATTTGC
*Alpl*	Forward: AACCCAGACACAAGCATTCC Reverse: GCCTTTGAGGTTTTTGGTCA
*Bglap*	Forward: ATCCAGAGCTGTGGCACACA Reverse: GGAAAGAAACGCCCGAAGA
*Col1a1*	Forward: TGGCGGTTATGACTTCAGCTT Reverse: CTCAAGGTCACGGTCACGAAC

*Runx2*, Runt-related transcription factor 2; *Alpl*, alkaline phosphatase; *Bglap*, bone gamma carboxyglutamate protein; *Col1a1*, collagen type I alpha 1 chain gene.

## Data Availability

The original contributions presented in this study are included in the article/Supplementary Materials. Further inquiries can be directed to the corresponding author.

## Supplementary Materials

The following supporting information can be downloaded at https://www.mdpi.com/article/10.3390/ijms27146489/s1. References [61,62,63,64] are cited in Supplementary Materials.
