# Supplementary material for: NLRC5 Deficiency Delays Bone Healing by Inhibiting Osteogenic Differentiation of Bone Marrow-Derived Stem Cells and Altering the Immune Microenvironment
Source: Int J Mol Sci. 2026 Jul 21;27(14):6489. doi: 10.3390/ijms27146489 (PMC13411262; doi:10.3390/ijms27146489)
Supplement: Supplementary file 1 [file ijms-27-06489-s001.zip › Supplementary Materials S1.pdf]

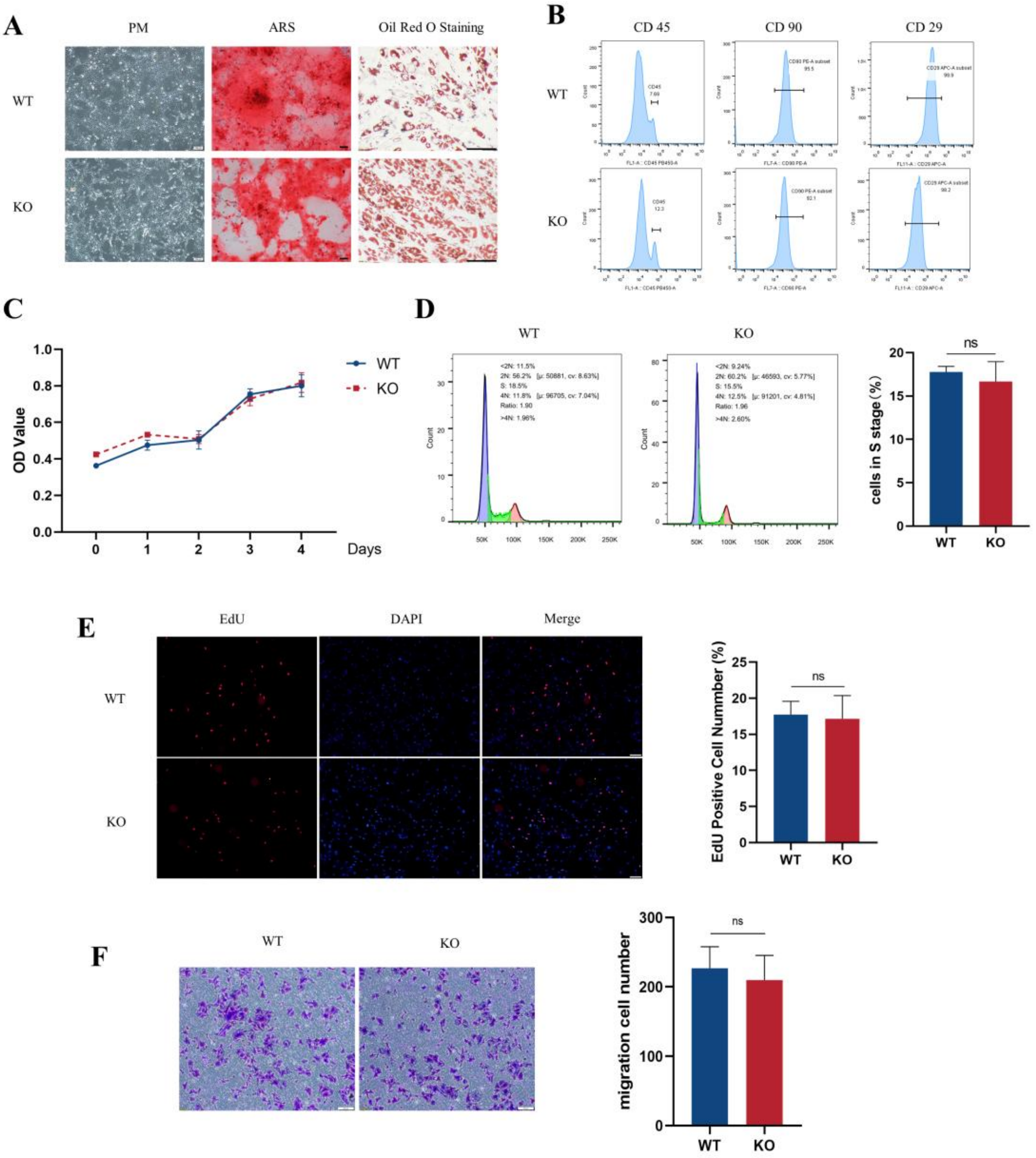

Supplement Figure. Isolation and characterization of BMSCs.

(A) Primary BMSCs were obtained on the third generation (left, scale = 200 μm), Alizarin red S staining (middle; scale = 200 μm), Oil Red O staining (right; scale = 200 μm). (B). Surface marker expression of BMSCs were assessed using flow cytometry. (C) Proliferative activity of BMSCs as detected by CCK-8 assay. (D) Cell cycle analysis. (E) EdU staining assay. (F) Transwell cell migration assay.

Representative cell counts:

		CD4+T	MDSCs	Neutrophils		Eosinophils	B cells			
	**sample**	**C04**	**C08**	**C14**	**C15**	**C20**	**C25**	**C26**	**C33**	**C34**
WT	**M01_C001**	227	20097	25125	10353	6982	1988	10844	1887	994
	**M01_C002**	262	21409	21868	9280	7863	2223	11602	1924	656
	**M01_C003**	366	22404	18417	6420	9916	2784	12966	2379	911
KO	**M01_C004**	617	12748	12974	4044	12821	2620	11970	3547	870
	**M01_C005**	626	10802	6116	1525	9484	2000	8719	2632	1007
	**M01_C006**	490	12649	11317	4172	8917	2364	10951	4084	1326

		Th2	Tfh	Treg	Th1	NaïveCD4 T
WT	**M01_C001**	17	39	17	13	10
	**M01_C002**	24	46	20	34	14
	**M01_C003**	41	53	14	29	15
KO	**M01_C004**	62	55	6	36	41
	**M01_C005**	96	46	12	45	50
	**M01_C006**	59	41	8	29	22
