# Supplementary material for: NLRC5 Deficiency Delays Bone Healing by Inhibiting Osteogenic Differentiation of Bone Marrow-Derived Stem Cells and Altering the Immune Microenvironment
Source: Int J Mol Sci. 2026 Jul 21;27(14):6489. doi: 10.3390/ijms27146489 (PMC13411262; doi:10.3390/ijms27146489)
Supplement: Supplementary file 1 [file ijms-27-06489-s001.zip › Supplementary Materials S2.pdf]

Detailed experimental procedures for RNA-seq and CyTOF

RNA-seq

The detailed methods for RNA-seq and data analysis are as follows: Total RNA was extracted from BMSCs, followed by library preparation according to the Illumina standard instruction (VAHTS Universal V6 RNA-seq Library Prep Kit for Illumina®). An Agilent 4200 bioanalyzer was employed to evaluate the concentration and size distribution of the cDNA library before sequencing with an Illumina NovaSeq 6000 platform. The protocol for high-throughput sequencing was fully performed according to the manufacturer's instructions (Illumina). Each sample was sequenced to a depth of approximately 20 million reads. The raw reads were filtered using Seqtk before mapping to the genome using Hisat2 (version 2.0.4). Gene fragments were counted using StringTie (version 1.3.3b), followed by TMM (trimmed mean of M values) normalization. Significant differentially expressed genes (DEGs) were identified as those with a false discovery rate (FDR) < 0.05 and a fold-change > 2 using edgeR software.

CyTOF

Preparation of cell suspensions

Single-cell suspensions were prepared as follows. Bone marrow cells were collected by flushing the entire marrow cavity of femurs and tibiae from 4-week-old WT and KO mice. The cell suspension was incubated with 3 volumes of red blood cell lysis buffer on ice for 15 minutes. After centrifugation at 400×g for 5 minutes at 4°C, the cells were washed once with PBS. The cell precipitates were resuspended in 5mL of pre-cooled fluorescence activated cell sorting (FACS) buffer (1×phosphate buffered saline (PBS) supplemented with 0.5% bovine serum albumin), and then centrifuged at 400×g for 5min at 4°C. The supernatant was discarded and the cell precipitates were resuspended in FACS buffer again. The number of cells was counted and the quality of samples for subsequent analysis should meet the following requirement: The

number of cells should not be less than 3×106 and the viability rate should be higher than 85%.

Antibodies

For mass cytometry analysis, purified antibodies were obtained from BioLegend, eBioscience, BioXcell, R&D systems and BD Biosciences using clones listed in Supplementary Table 1. Antibody labeling with the indicated metal tag was performed using the MaxPAR antibody Labelling kit (Fluidigm). Conjugated antibodies were titrated for optimal concentration before use.

Mass cytometry staining, data acquisition

Cells were washed once with 1xPBS and then stained with 100μL of 250nM cisplatin (Fluidigm) for 5min on ice to exclude dead cells, and then incubated in Fc receptor blocking solution before stained with surface antibodies cocktail for 30 min on ice. Cells were washed twice with FACS buffer (1xPBS+0.5%BSA) and fixed in 200μL of intercalation solution (Maxpar Fix and Perm Buffer containing 250nM 191/193Ir, Fluidigm) overnight. After fixation, cells were washed once with FACS buffer and then perm buffer (eBioscience), stained with intracellular antibodies cocktail for 30 min on ice. Cells were washed and resuspend with deionized water, adding into 20% EQ beads (Fluidigm), acquired on a mass cytometer (Helios, Fluidigm).

Flow cytometric gating strategy

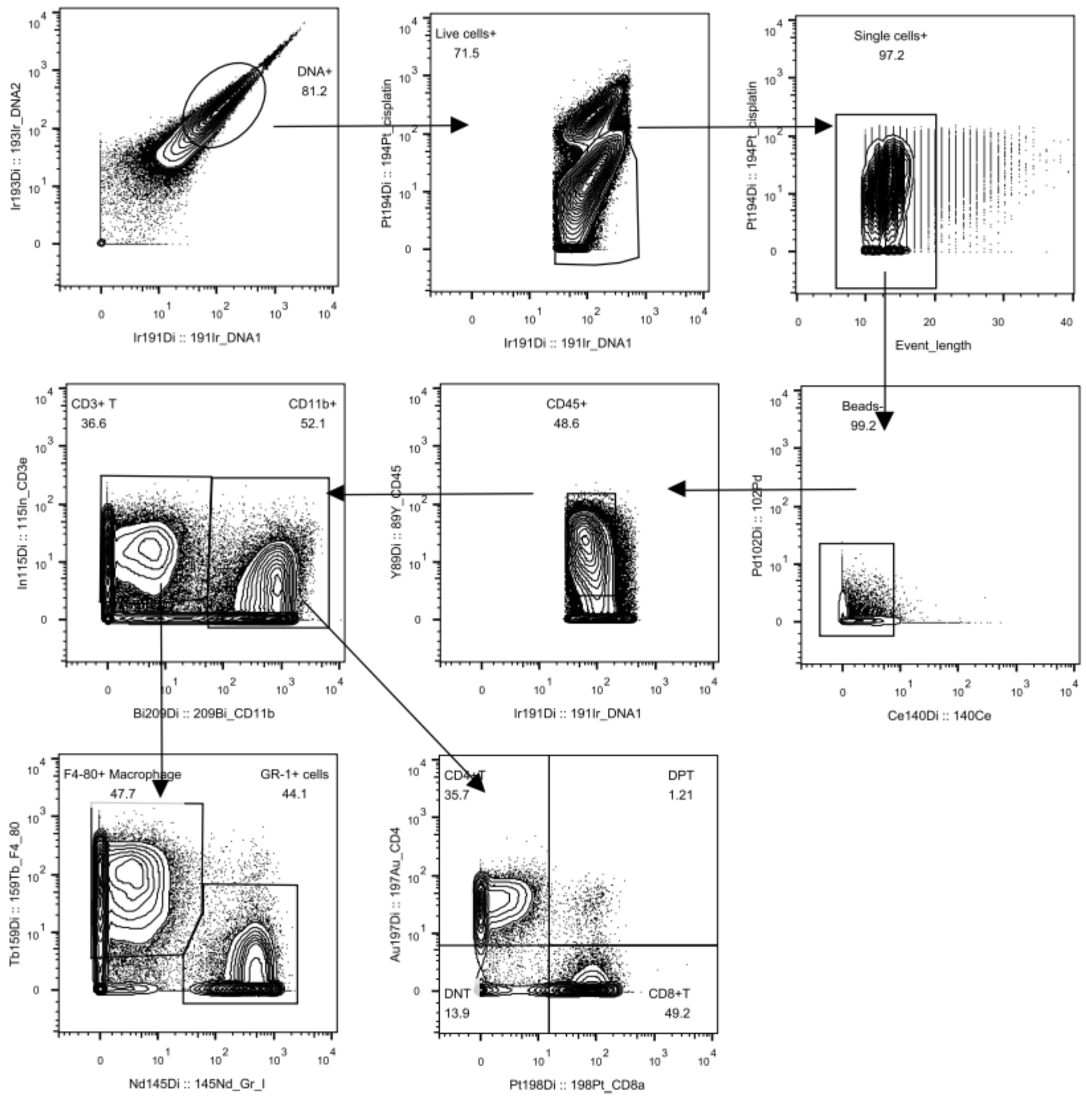

CyTOF data analysis

1. Data of each sample were debarcoded from raw data using a doublet-filtering scheme [1] with unique mass-tagged barcodes.
2. Each .fcs file generated from different batches were normalized through bead normalization method [2].
3. Manually gate data using a FlowJo software to exclude to debris, dead cells and doublets, leaving live, single immune cells.
4. Apply the X-shift clustering algorithm [3] to all cells to partition the cells into distinct phenotypes based on marker expression levels.
5. Annotate cell type of each cluster according to its marker expression pattern on a heatmap of cluster vs marker.
6. Use the dimensionality reduction algorithm t-SNE [4] to visualize the high-

dimensional data in two dimensions and show distribution of each cluster and marker expression and difference among each group or different sample type.

7. Perform T-test statistical analysis on the frequency of annotated cell population.

References:

1. Zunder, E.R., Finck, R., Behbehani, G.K., Amir, A.D., Krishnaswamy, S., Gonzalez,V.D., Lorang, C.G., Bjornson, Z., Spitzer, M.H., Bodenmiller, B., et al. (2015). Palladium-based mass tag cell barcoding with a doublet-filtering scheme and single-cell deconvolution algorithm. Nat. Protoc. 10, 316–333.

2. Finck, R., Simonds, E.F., Jager, A., Krishnaswamy, S., Sachs, K., Fantl, W., Pe'er, D., Nolan, G.P., and Bendall, S.C. (2013). Normalization of mass cytometry data with bead standards. Cytometry A 83, 483–494.

3. Samusik, N., Good, Z., Spitzer, M. H., Davis, K. L. & Nolan, G. P. (2016). Automated mapping of phenotype space with single-cell data. Nat. Methods 13, 493–496.

4. van der Maaten, L., and Hinton, G. (2008). Visualizing data using t-SNE. J. Mach. Learn. Res. 9, 2579–2605.

Reagents:

Reagents	Cat #	Vender
Bovine Serum Albumin	V900933	Sigma-Aldrich
Maxpar Fix and Perm Buffer	201067	Fluidigm
Cell-ID Cisplatin-194Pt	201194	Fluidigm
Cell-ID Intercalator-Ir	201192B	Fluidigm
EQ Four Element Calibration Beads	201078	Fluidigm

yangxiangshu@plttech.com

Zhejiang Puluoting Health Technology Co., Ltd. (PLT Tech Co., Ltd., PLT Tech)

14th Floor, Building 5, No.1500 Wenyi West Road, Hangzhou, China, 311121
